# Immune approaches to the treatment of breast cancer, around the corner?

**DOI:** 10.1186/bcr3620

**Published:** 2014-02-25

**Authors:** Carmen Criscitiello, Angela Esposito, Lucia Gelao, Luca Fumagalli, Marzia Locatelli, Ida Minchella, Laura Adamoli, Aron Goldhirsch, Giuseppe Curigliano

**Affiliations:** 1 Division of Early Drug Development for Innovative Therapies, Istituto Europeo di Oncologia, Via Ripamonti 435, 20133 Milano, Italia

## Abstract

Immunotherapy for the treatment of breast cancer can be categorized as either (a) specific stimulation of the immune system by active immunization, with cancer vaccines, or (b) passive immunization, such as tumor-specific antibodies (including immune modulators) or adoptive cell therapy that inhibit the function of, or directly kill, tumor cells. We will present the current information and the future perspectives of immunotherapy in patients with breast cancer, including the prognostic role of tumor infiltrating lymphocytes, immune signatures, targeted therapies modulating the immune system, and tumor antigen cancer vaccines. Active immunotherapy in breast cancer and its implementation into clinical trials have been largely a frustrating experience in the last decades. The concept that the immune system regulates cancer development is experiencing a new era of interest. It is clear that the cancer immunosurveillance process indeed exists and potentially acts as an extrinsic tumor suppressor. Also, the immune system can facilitate tumor progression by sculpting the immunogenic phenotype of tumors as they develop. Cancer immunoediting represents a refinement of the cancer immunosurveillance hypothesis and resumes the complex interaction between tumor and immune system into three phases: elimination, equilibrium, and escape. Major topics in the field of immunology deserve a response: what do we know about tumor immunogenicity, and how might we therapeutically improve tumor immunogenicity? How can we modulate response of the immune system? Is there any gene signature predictive of response to immune modulators? The success of future immunotherapy strategies will depend on the identification of additional immunogenic antigens that can serve as the best tumor-rejection targets. Therapeutic success will depend on developing the best antigen delivery systems and on the elucidation of the entire network of immune signaling pathways that regulate immune responses in the tumor microenvironment.

## Introduction

**Figure 1:**
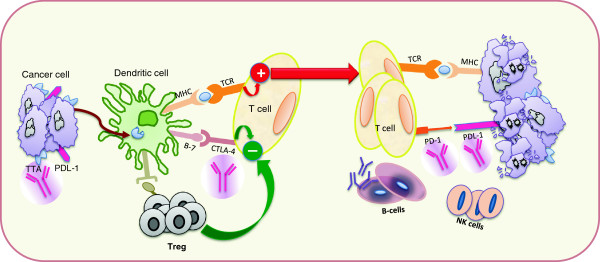
**Immune system functions and components relevant to breast cancer therapy.** CTLA-4, cytotoxic T lymphocyte-associated antigen 4; MHC, major histocompatibility complex; NK, natural killer; PD-1, programmed death-1; PDL-1, PD-1 ligand 1; TAA, tumor-associated antigen; TCR, T-cell receptor; Treg, regulatory T.

Evading immune destruction is an emerging hallmark of cancer. The immune system plays a dual role in cancer: it not only can suppress tumor growth by destroying cancer cells or inhibiting their outgrowth but also promotes tumor progression either by selecting for tumor cells that are more fit to survive in an immunocompetent host or by establishing conditions within the tumor microenvironment that facilitate tumor outgrowth. The conceptual framework called ‘cancer immunoediting’ integrates the immune system’s dual host-protective and tumor-promoting roles. Nonetheless, numerous studies have shown that tumors can be recognized and contained for extended periods of time by the immune response through the concerted action of the innate and adaptive immune responses [1]. Despite these efforts, cancer still develops, at increased frequency with age, as a consequence of selecting less immunogenic tumor cells (immunoediting) or the increased effectiveness of tumor-mediated immunosuppression (immune subversion) or both [2,3]. In Figure 1 are reported major functions and components of the immune system relevant for potential breast cancer (BC) therapy.

In BC, recent evidence has demonstrated that immune-related factors play an important role in defining patient prognosis and their response to treatment. These include the extent of lymphocyte infiltration in tumor tissue and a class of gene expression signatures, both of which have the potential to more precisely define patients’ clinical evolution and identify patient subgroups with different sensitivities to standard treatments. Despite these new insights, clinicians still rely primarily on classic clinical-pathological features such as tumor size and lymph node involvement for daily patient management, and it is difficult to see how these parameters may be implemented in the clinic in the future. This review will highlight the importance of exploring the immune system in both research and clinical settings, since its role in defining BC behavior is proving to be a significant factor.

## The role of the lymphocytic infiltrate in breast cancer

Over the past few decades, a growing body of evidence has emerged demonstrating that the immune system participates both in tumor development (via chronic inflammation orchestrated by the innate immune system) and in tumor elimination and control (through the actions of the adaptive immune system) [4]. The presence of tumor-infiltrating lymphocytes (TILs) is observed in some breast tumors and has been reported to be a good prognosis feature for some forms of the disease [5-7], particularly for rapidly proliferating tumors correlating with negative axillary nodal status, smaller tumor size, and lower grade [6]. Similarly, TIL count has been associated with better survival in patients with estrogen receptor (ER)-negative tumors [8,9]. Also, TILs have been negatively correlated with patient’s age at diagnosis [8,9]. Recently, TILs have emerged as a potential prognostic and predictive marker in BC, especially in the triple-negative (TN) and HER2-positive subtypes. Loi and colleagues [10] have evaluated the predictive value of TIL in 935 patients in the FinHER (Finland Herceptin) trial. Among the 134 TN patients receiving docetaxel and fluorouracil/epirubicin/cyclophosphamide (FEC) or vinorelbine and FEC, the 3-year recurrence-free survival was 90% in the case of extensive lymphocyte infiltrate (intratumoral or stromal TIL >30%) versus 66% in the case of non-extensive lymphocyte infiltrate (*P* = 0.007). In locally advanced BC treated with neoadjuvant chemotherapy, the presence of TILs in the primary biopsy predicts pathologic complete response [9]. In this study, TIL was composed of both CD3^+^ and CD20^+^ cells. The same group has recently evaluated the prognostic and predictive value of TILs in a large cohort of lymph node-positive early-BC patients prospectively randomly assigned to receive either high-dose anthracycline-based chemotherapy or a combinatorial regimen involving anthracyclines and docetaxel within the BIG02-98 trial [11]. TILs^+^ were strongly associated with good prognosis among patients with TN BC, whereas TILs had no prognostic value among patients with HER2^+^ BC. However, TILs^+^ were associated with improved therapeutic responses to high-dose anthracyclines in patients with HER2^+^ BC [11]. Interestingly, Demaria and colleagues [12] have reported that taxane-based primary chemotherapy converted 7 out of 21 breast tumors from TIL^−^ to TIL^+^ and that the post-chemotherapy TIL^+^ status was associated with an improved clinical response. Furthermore, Ladoire and colleagues [13] have reported that neoadjuvant chemotherapy increased the CD8^+^ infiltration in the tumor. This post-treatment infiltration is associated with an improved outcome. It is worthy to highlight that one of the proposed mechanisms of action of chemotherapy is the induction of an anti-tumoral immune response. The group led by Zitvogel [14] has reported that cytotoxic agents, including anthracyclines, oxaliplatin, and radiation therapy, induce immunogenic cell death through high-mobility-group box 1 release. In this model, chemotherapy kills cancer cells. This modality of cell death itself induces signal danger and tumor-specific T-cell response. Interestingly, the induction of T-cell response is highly heterogeneous according to individuals and tumor characteristics. This inter-individual variability in the ability of chemotherapy to induce immune response could explain why chemotherapy is not working the same in all patients. Overall, these findings provide evidence [13] that TIL assessed at baseline could stratify patients into a high- or low-risk population. Moreover, small retrospective hypothesis-generating studies suggested that post-chemotherapy lymphocyte infiltration at the tumor bed may be correlated with prognosis. In conclusion, TIL^+^ BCs present specific features that could have specific clinical implications in terms of prognosis. These data from population-based studies or randomized trials will need additional validation using a prospective trial before implementation. A potential implication could be to use TILs as inclusion criteria in large adjuvant trials that include TN BC or HER2-positive BC. Another potential prognostic implication relates to the potentially good prognosis of patients who were TIL^−^ at baseline but became TIL^+^ after neoadjuvant therapy [12]. Nevertheless, if these data are confirmed, they could be used to better stratify which patients should be included in post-neoadjuvant trials. Finally, the information that TIL^+^ is associated with good outcome is an argument to develop immune strategies in patients with BC. TIL assessment could identify a subset of TIL^−^ TN and HER2^+^ BC patients who deserve additional therapy. Genomic predictor of chemo-immunization could represent a new prognostic parameter that will allow clinicians to select which TIL^−^ BCs are candidates for new therapeutics in the (neo)adjuvant setting, thus decreasing toxicity, decreasing cost, and making feasible (neo)adjuvant trials. TILs are represented mainly by non-activated T cells which often become activated after exposure to chemotherapy. More recently, the nature of TILs has been better characterized. Ruffell and colleagues [15] have reported that TILs are composed mainly of CD3^+^/CD56^−^ T cells but that a minority consisted of natural killer (NK) cells or CD20^+^ cells. The majority of CD3^+^ cells were either CD4^+^ or CD8^+^ T cells. Interestingly, CD8^+^ cells did not express Granzyme B at baseline, which means that they did present inactivation status, but they did express Granzyme B after neoadjuvant chemotherapy in one third of the patients. Lastly, a minority of TILs presented T and NK cell features [15]. The genomic characteristics of TIL^+^ tumors are important to understand which molecular mechanisms lead to lymphocyte infiltration. Genomic instability may promote anti-tumor immune response through tumor-associated antigens. Some mechanisms of chemokine release by the tumor have been described and correlated with lymphocyte attraction. TILs have been associated with CXCL9 and CXCL13 expression by the tumor [9]. TIL^+^ tumors present a specific methylation pattern on immune-related genes, including CCL5 [16]. A cluster of chemokines is lost in a subset of BC [17].

## Immune-related gene signatures

In past years, gene expression profiling has been used in an effort to more precisely define BC taxonomy and identify prognostic and predictive signatures [18]. The common denominator between the majority of the ‘first generation’ signatures is their overall capacity to detect subtle differences in the cell cycle and proliferation. For this reason, they have not been found to be prognostic in the TN or HER2^+^ subtypes since these tumors are by ‘nature’ highly proliferative. Several investigators have tried to overcome the limitations of these ‘first generation’ signatures by focusing on the BC microenvironment or immune response (or both) to define promising ‘second generation’ prognostic signatures (Table 1) [19]. Unsupervised gene expression profiling of cancer-associated stroma revealed a signature enriched for CD8^+^ T-cell responses that was predictive of good prognosis [20]. An immune response module, the STAT1 module, has been shown to be associated with survival in patients with TN and HER2^+^[21,22], and in the same BC subtypes, the overexpression of immune-related genes was able to identify subgroups of patients with a better prognosis [7,23]. Together, these investigations suggest that immune modulation may be important, particularly in highly proliferative subtypes such as TN and HER2^+^.

**Table 1:** Immune signatures and their development

**Immune signature**	**Signature development**
Immune response (IR) module [23]	A subclass of estrogen receptor-negative (ER^−^) tumors that overexpress IR genes and that have a good prognosis compared with the rest of ER^−^ breast tumors independently of lymph node status or lymphocytic infiltration was identified. Subsequently, an associated module of complement and IR genes that define prognostic markers was identified and validated in over 240 ER^−^ samples.
STAT1 module [22]	On the basis of the literature, genes to act as ‘prototypes’ for different biological processes - ER for ER signaling, HER2 for HER2 signaling, AURKA for proliferation, CASP3 for apoptosis, VEGF for angiogenesis, PLAU for tumor invasion/metastasis, and, in this case, STAT1 for immune response - were selected. A comparison of linear models was then applied to generate modules of genes specifically associated with each of the prototype genes but not with the other prototypes.
B-cell metagene [7]	Gene expression patterns of 200 patients who did not receive systemic treatment and co-regulated genes related to proliferation, steroid hormone receptor expression, and B-cell and T-cell infiltration were identified after hierarchical cluster analysis was performed. Metagenes were calculated as a surrogate for all genes contained within a particular cluster and their expression was correlated to time to metastasis. The B-cell metagene showed independent prognostic information in carcinoma with high proliferative activity.
IgG, HCK, MHC-I, MHC-II, LCK, STAT1, and IFN metagenes [24]	Unsupervised hierarchical clustering of genes in 12 primary invasive breast cancer datasets as well as combined datasets revealed a large cluster of genes with functions in immune cells. Among this cluster, clusters that contained a minimum number of elements and a minimal average correlation were selected, and seven metagenes were derived. Each metagene then was associated with a cell type or immunological state or both.
HR^neg^/T^neg^ signature [25]	A cohort of patients with node-negative, adjuvant treatment-naïve hormone receptor-negative (HR^neg^), and triple-negative (T^neg^) breast cancer has been used to define and validate genes predictive for distant metastatic relapse. A composite HR^neg^/T^neg^ signature index was able to identify cases likely to remain free of metastatic relapse with high accuracy. Of note, significant positive correlation was observed between the HR^neg^/T^neg^ index and three independent immune-related signatures (STAT1, IFN, and IR), and network analysis showed that the signature was linked to immune/inflammatory cytokine regulation.
Support Vector Machine (SVM) classifier [26]	Gene expression data of 2,145 invasive early breast adenocarcinomas were collected and used to test and validate the predictive performance of an SVM classifier based on a 368-gene expression signature associated with medullary breast carcinoma (MBC), which displays a basal profile but has good prognosis. The SVM model accurately classified all MBC samples in the learning and validation sets and was able to separate 466 cases of basal breast cancers into two subgroups (subgroup 1 and subgroup 2) containing, respectively, good- and poor-prognosis tumors. Ontology analysis revealed, among other features, effective IR in the good-prognosis subgroup.

*AURKA*, aurora kinase A; *CASP3*, caspase 3; *HCK*, hemopoietic cell kinase; *IFN*, interferon; *IgG*, immunoglobulin G; *LCK*, lymphocyte-specific kinase; *MHC*, major histocompatibility complex; *PLAU*, Urokinase-type plasminogen activator; *STAT1*, signal transducer and activator of transcription 1; *VEGF*, vascular endothelial growth factor.

## Immunogenicity and response to therapies affecting the immune system

BC has not been traditionally considered immunogenic, as it does not occur at a higher incidence in the immunosuppressed populations who have been treated with immunosuppressive therapies [27], as opposed to melanoma and renal cell carcinoma, which have been traditionally considered more responsive to immunotherapies. However, it seems that, despite a weak influence on primary tumor growth, the immune system is effective in preventing BC metastases [28-30]. The heterogeneous expression of tumor antigens within the primary tumor or its metastases, the modification of antigenic profile during the tumor progression, and the low levels of the antigen, major histocompatibility complex proteins, and other co-stimulatory proteins necessary to generate a strong immune response can explain this low immunogenicity. Moreover, the tumor microenvironment releases immune-suppressive factors that make the antigen presentation difficult and that have a negative impact on the immune response [31]. Moreover, by blocking endogenous immune checkpoints that normally terminate immune responses after antigen activation, it is possible to evade immune destruction. Besides the example of anti-cytotoxic T lymphocyte-associated antigen 4 (CTLA-4) antibody (ipilimumab) for the treatment of patients with advanced melanoma [32-34], programmed death 1 (PD-1) might extend the spectrum of immunotherapy clinical activity in tumor types traditionally not considered to be immunogenic, such as metastatic non-small-cell lung cancer [35]. Nonetheless, it seems that any tumor could be immunogenic with appropriate immune activation.

On the other hand, activation of the immune system could mediate the anti-tumor effects of several anti-cancer drugs. In a neoadjuvant clinical trial (Trial of Principle (TOP) study) in which patients with ER^−^ BCs were treated with anthracycline monotherapy, high immune module scores were associated with sensitivity to anthracyclines [36]. The immune system seems also to be pivotal in determining the response to monoclonal antibodies (mAbs) and tyrosine kinase inhibitors, and some evidence indicates a possible role in the response to endocrine treatment. The humanized immunoglobulin (IgG1) mAb trastuzumab is commonly used to treat patients with HER2^+^ BC with increased response rate and survival [37,38]. Antibody-dependent cellular cytotoxicity (ADCC) has long been implicated as one of the mechanisms of action for trastuzumab [39,40]. Correlative studies have suggested that patients responding to mAb treatment had higher *in situ* infiltration of leukocytes and increased capacity to mediate ADCC activity. Tumor regressions *in vivo* due to anti-HER2 mAb therapy also require an effective adaptive anti-tumor immune response to reach optimal therapeutic effects, and levels of CD8 and interferon-gamma have been shown to correlate with anti-HER2 treatment. Therefore, complete tumor response after molecular targeted therapies requires a functioning immune system, pointing the way toward radically new combination therapies with a targeted and immune approach [41].

In recent years, the better knowledge of BC biology has provided an opportunity to develop some types of immunotherapy to overcome the relative non-immunogenic property of BC and improve immune response. Some molecules such as PD-1 and its ligand PDL-1, CTLA-4, and immune cells such as regulatory T (Treg) cells are involved in the induction of tolerance to antigens, and their upregulation is associated with increased risk of developing BC [42]. The mAbs against antigen tumor target or immune-regulatory molecules, cell-based therapies including adoptive transfer of *ex vivo*-activated T cells and NK cells, or blockade of Treg cells could be useful to amplify the anti-tumor response. Forkhead box P3 (FOXP3)^+^ Treg cells are crucial for the induction and maintenance of peripheral tolerance to self-antigens. While exerting their function, Treg cells can also suppress immune responses to tumor antigens, alloantigens, and allergens [5,43]. The prognostic importance of FOXP3 expression in patients with BC has been investigated. FOXP3 expression in BC was associated with worse overall survival probability, and the risk increased with increasing FOXP3 immunostaining intensity [44]. FOXP3 was also a strong prognostic factor for distant metastases-free survival but not for local recurrence risk. In multivariate analysis, FOXP3 resulted in an independent prognostic factor, and the hazard ratios of FOXP3 expression and of lymph node positivity were similar. In the Milan 3 trial, the probability rates of 10-year survival in the node-negative subgroup were 100% for FOXP3-negative and 82% for FOXP3-positive patients; corresponding rates in the node-positive subgroup were 82% for FOXP3-negative and 41% for FOXP3-positive patients. According to these data, Treg cells may play an important role in BC immunopathology because of their potent suppressive activity of both T-cell activation and effector function. The modulation of the immune response by Treg cells can enhance immune response and improve patients’ clinical outcome. An alternative may be targeting the immune checkpoint molecules. Preclinical models have shown that inhibitory signals mediated by co-receptors on tumor-specific T cells impede anti-tumor immunity and suggest that blockade of such activity may aid host immune-mediated tumor elimination. Recently, T-cell checkpoint inhibitors were shown to induce durable tumor regression and stabilization of disease in patients with advanced cancer and give new hope for the treatment of patients with BC [45]. PD-1 is an inhibitory receptor expression on activated T and B cells whose activity may suppress anti-tumor immunity. Phase I data [46] showed that blocking the PD-1 immune checkpoint with a targeted antibody may be safe and well tolerated with evidence of anti-tumor activity, presumably through immunogenic mechanisms as PD-1 receptors are not expressed on non-hematologic malignancies. The combinatorial anti-tumor effects of anti-HER2 and anti-PD1 demonstrated in experimental models are important proof-of-principle experiments that the anti-tumor adaptive immunity provoked by anti-HER2 can be capitalized upon, providing a new model of potential combination treatment for women receiving trastuzumab.

Another interesting immune molecule is CTLA-4 (CD152), which is similar to PD-1, but its immune-inhibitory signals are different. CTLA-4 knockout mice display early lethality, unlike PD-1 knockouts, which demonstrate late-onset and organ-specific autoimmunity. Anti-CTLA4 mAb treatment has shown robust tumor responses in phase III trials, but with considerable adverse events [32]. Still, combining anti-CTLA-4 mAb with trastuzumab has shown synergy in preclinical mouse models [47].

Hence, immunotherapeutics that augment CD8 T-cell anti-tumor activity - such as anti-PD1 and anti-CTLA4 mAbs - given in combination with trastuzumab in patients with HER2^+^ BC may improve outcome by involving and enhancing critical host immunity [41,48,49].

Given this evidence, the evaluation of baseline immune response and the identification of easy-to-define surrogate markers of immune system activation could be helpful in the management of BC to identify patients who may benefit from these combination therapies, even eliminating the need for combination cytotoxic chemotherapy.

## Vaccine-based therapies for breast cancer

Vaccines constitute an active and specific immunotherapy designed to stimulate the intrinsic anti-tumor immune response by presenting tumor-associated antigens (TAAs) expressed on normal tissues that are overexpressed on tumor cells. Malignant cells can express both normal self-antigens and specific TAAs that arise from genetic mutations or epigenetic changes or both, recognized by the immune response through either their loss or *de novo* aberrant expression. Many TAAs (including MUC1, HER-2, CEA, hTER, and WT1) have been identified and been shown to be specifically recognized by T cells [50]. Induction of strong immunity by cancer vaccines is expected to lead to the establishment of immunological memory, thereby preventing tumor recurrence.

The identification of immunological and genetic features affecting immune response in patients with minimal tumor burden represents the optimal background for development of clinical studies in the adjuvant setting as it should allow the immune system to mount a response before it is overtaken. An active immunization may provide a non-toxic therapeutic modality able to induce anti-tumor immune responses in patients with cancer [51]. However, the majority of the trials so far have been conducted in the metastatic setting, which could have significantly impacted the results because of the large tumor burden [52-56]. Cancer vaccines are more effective when given in combination with standard cancer treatments, which appear to increase their effectiveness [57-59]. The elimination of Treg cells potentially provides the basis [55] for the synergistic effect between cancer vaccines and chemotherapy [58]. To optimize the immunological response to a vaccination strategy, it is paramount to identify both the target antigen and the patient population to be targeted. Large population analyses on specific BC subtypes are necessary to select patients who have higher probability to express that specific antigen. To improve immunotherapy trials, investigators have to take into account the ability to initiate tumor-specific immunity, either directly by providing tumor-associated antigens or indirectly by favoring the cross-presentation of endogenous tumor antigens; the capacity to recruit effector immune cells within the tumor site, by increasing tumor visibility; and the ability to preserve immune cell functionality within the tumor microenvironment through the subversion of immune-escape mechanisms. In the adjuvant setting, we can propose several surrogate markers of activity of a vaccination strategy. Some are related to immunomonitoring approaches; some others can be related to detection of disseminated tumor cells in the bone marrow or circulating tumor cells in the peripheral blood or both [60,61]. Combined therapies should be proposed to accomplish these three features. Furthermore, immunotherapy needs a long time before turning into an efficient anti-tumor response; that is why trials should ideally target well-defined patients in adjuvant settings [62].

## Conclusions

We have provided an overview of the recent evidence demonstrating that the immune system plays a critical role in defining BC prognosis and response to different treatments. However, more studies are necessary to precisely define the nature and role of TILs, establish the clinical value of immune-based prognostic and predictive gene signatures, and optimize standard treatments to effectively work in tandem with the anti-tumor immune response. Several questions are raised by all remarkable data presented in this review; answers to these questions should be considered possible areas of research in the following years. Should all patients with cancer be treated with an active immunotherapy approach or only individuals potentially more ‘responding’? How can we predict that the individual will develop an immune response against a particular antigen used in the vaccine formulation? Is there any genetic signature predicting response to immunotherapy? What are the risks associated with such a vaccination (that is, the possibility to develop an autoimmune response)? What is the durability of immune protection? Can we combine vaccine therapy with therapeutic mAbs or small target oriented molecules? What is the potential effect of antibodies modulating the immune system in patients with metastatic BC? Continued basic research into the molecular mechanisms regulating carcinogenesis and immunosurveillance/tolerance will identify new potential targets, introducing vaccine therapy in prevention trials for patients at high risk for developing cancer. A better understanding of the dialogue between cancer and immune cells could help to improve immunotherapeutic approaches and foster the development of new drugs that increase the ability of the immune response to provide effective and sustained anti-tumor immunity.

## Note

This article is part of a series on "*Recent advances in breast cancer treatment and the translational research behind them*", edited by Jenny Chang. Other articles in this series can be found at http://breast-cancer-research.com/series/treatment.

## Abbreviations

ADCC: Antibody-dependent cellular cytotoxicity; BC: Breast cancer; CTLA-4: Cytotoxic T lymphocyte-associated antigen 4; ER: Estrogen receptor; FEC: Fluorouracil/epirubicin/cyclophosphamide; FOXP3: Forkhead box P3; mAb: monoclonal antibody; NK: Natural killer; PD-1: Programmed death-1; TAA: Tumor-associated antigen; TIL: Tumor-infiltrating lymphocyte; TN: Triple-negative; Treg: Regulatory T.

## Competing interests

The authors declare that they have no competing interests.
